# Supplementary material for: Alternative Splicing in TRPA1 Drives Sensory Adaptation to Electrophiles in Drosophilids
Source: bioRxiv. 2025 May 15:2025.05.09.653172. Preprint. [Version 2] doi: 10.1101/2025.05.09.653172 (PMC12132172; doi:10.1101/2025.05.09.653172)
Supplement: Supplement 2 [file NIHPP2025.05.09.653172v2-supplement-2.pdf]

bioRxiv preprint doi: https://doi.org/10.1101/2025.05.09.653172; this version posted May 15, 2025. The copyright holder for this preprint (which was not certified by peer review) is the author/funder, who has granted bioRxiv a license to display the preprint in perpetuity. It is made available under aCC-BY 4.0 International license.

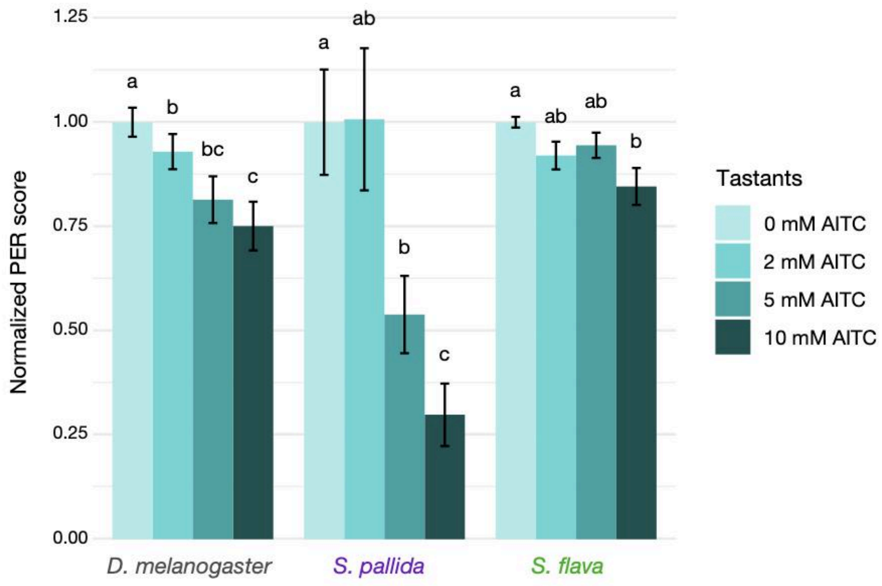

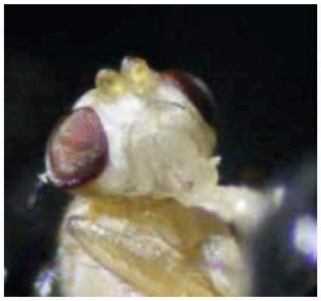

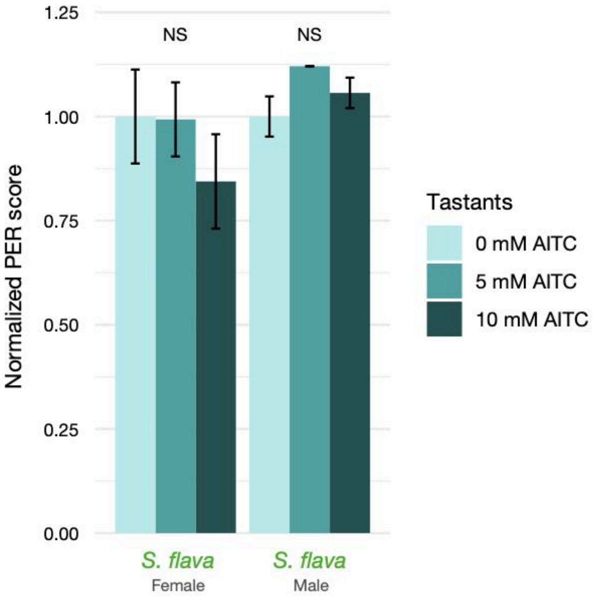

Supplementary Figure S1. Additional data of PER assay. (Top) Results of PER assay using male flies from the three focal species (n = 20-32 per treatment). (Bottom left) A photograph of *S. flava* female with their antennal segments (3rd and 4th) and maxillary palps removed. (Bottom Right) Results of PER assay in antenna/palp-less *S. flava* (n = 9-14 per treatment). For both PER assays, individual PER scores were normalized to the average score at 0 mM AITC. Differences in PER scores between tastants within species or sexes were analyzed by pairwise Wilcoxon rank-sum test with Benjamini-Hochberg correction ($p < 0.05$).

bioRxiv preprint doi: https://doi.org/10.1101/2025.05.09.653172; this version posted May 15, 2025. The copyright holder for this preprint (which was not certified by peer review) is the author/funder, who has granted bioRxiv a license to display the preprint in perpetuity. It is made available under aCC-BY 4.0 International license.

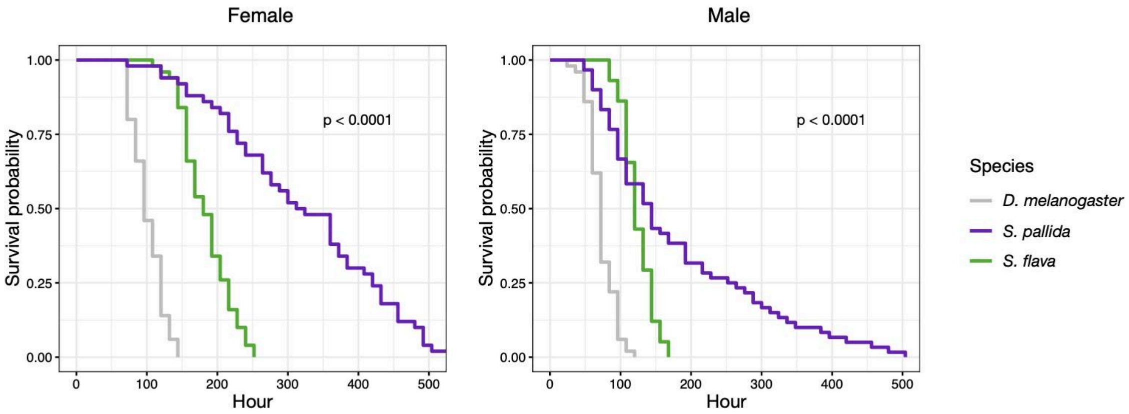

Supplementary Figure S2. Hunger tolerance assay of adult flies. Survival plots of (Left) female and (Right) male flies, each of which consists of n = 5-6 independent groups of 8-10 flies. Differences between species were analyzed by log-rank test. Only group-wide *p*-values were shown.

bioRxiv preprint doi: https://doi.org/10.1101/2025.05.09.653172; this version posted May 15, 2025. The copyright holder for this preprint (which was not certified by peer review) is the author/funder, who has granted bioRxiv a license to display the preprint in perpetuity. It is made available under aCC-BY 4.0 International license.

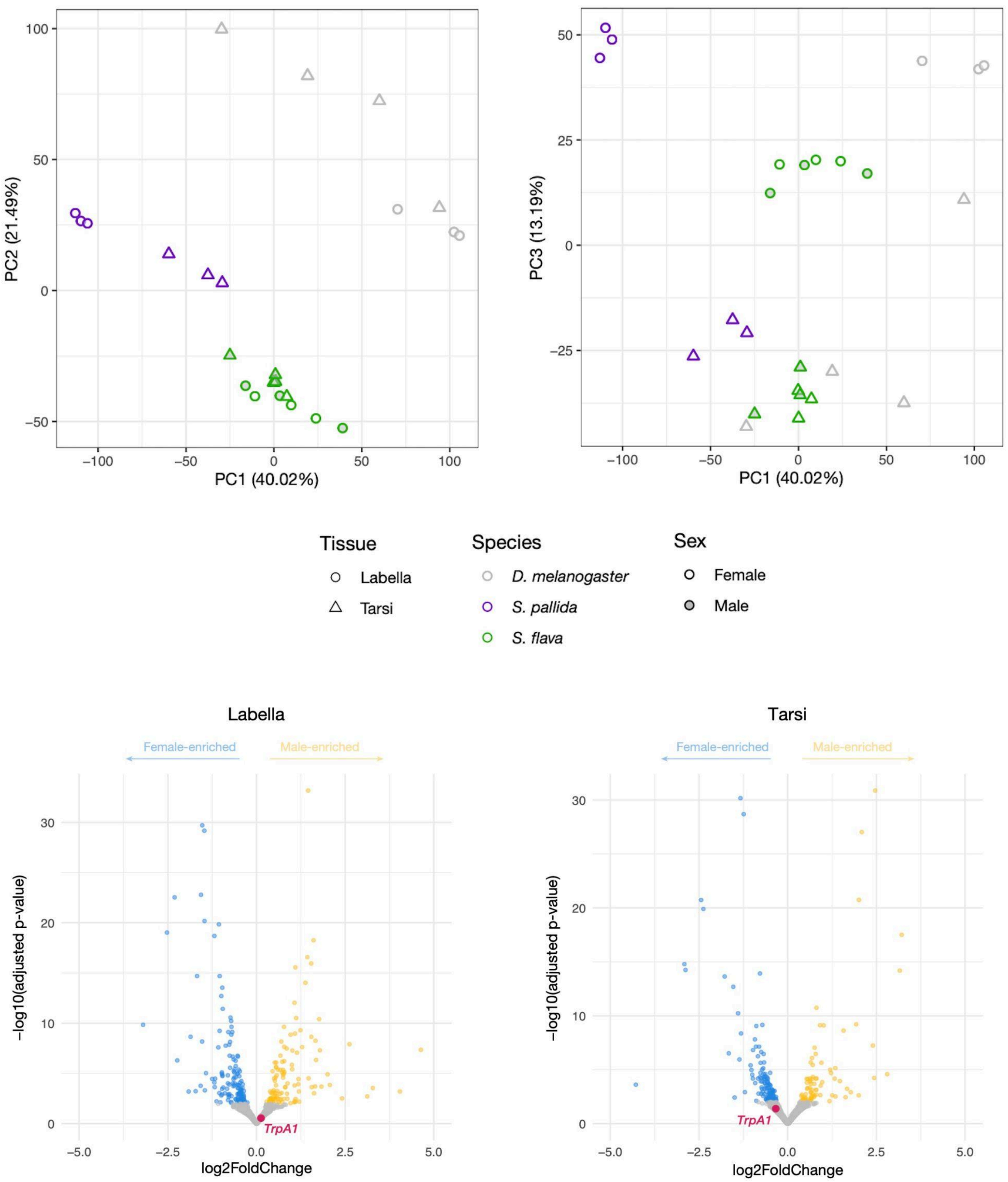

Supplementary Figure S3. Labellar and tarsal transcriptome analyses. (Top) PCA plots for 25 transcriptome datasets based on read count data of 8,920 orthologous genes. (Bottom) Volcano plots of DEG analysis between female and male *S. flava*. *TrpA1* was not differentially expressed between sexes in both labella and tarsi.

bioRxiv preprint doi: https://doi.org/10.1101/2025.05.09.653172; this version posted May 15, 2025. The copyright holder for this preprint (which was not certified by peer review) is the author/funder, who has granted bioRxiv a license to display the preprint in perpetuity. It is made available under aCC-BY 4.0 International license.

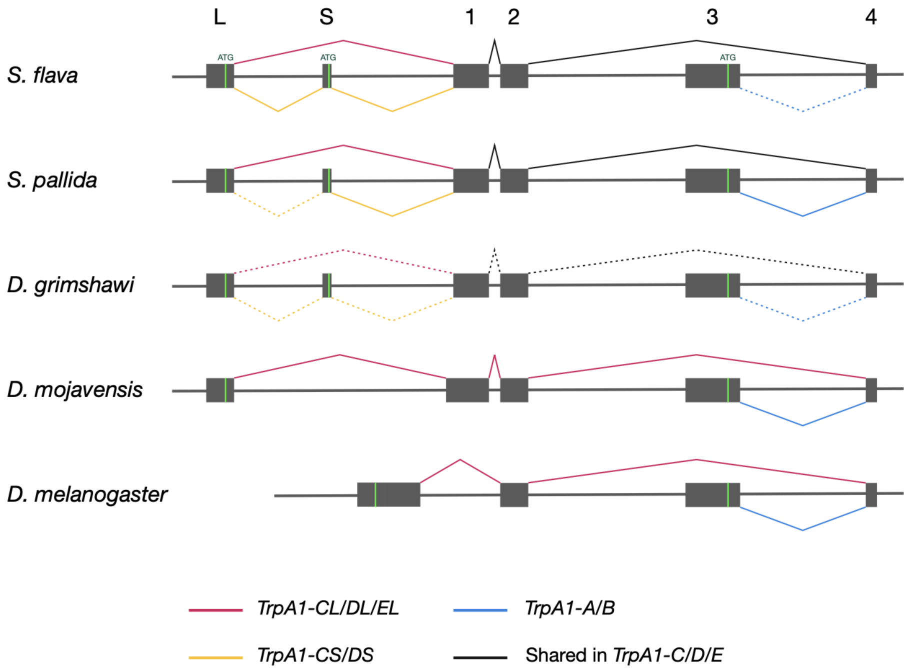

Supplementary Figure S4. Evolution of N-terminal exons of *TrpA1* across drosophilids. Colored lines indicate exon-intron boundaries specific to each isoform type. Dashed lines show the lack of transcriptional evidence (inferred only from genomic/CDS-level information). Green vertical lines indicate putative (i.e., the most upstream) translation start sites for each isoform.

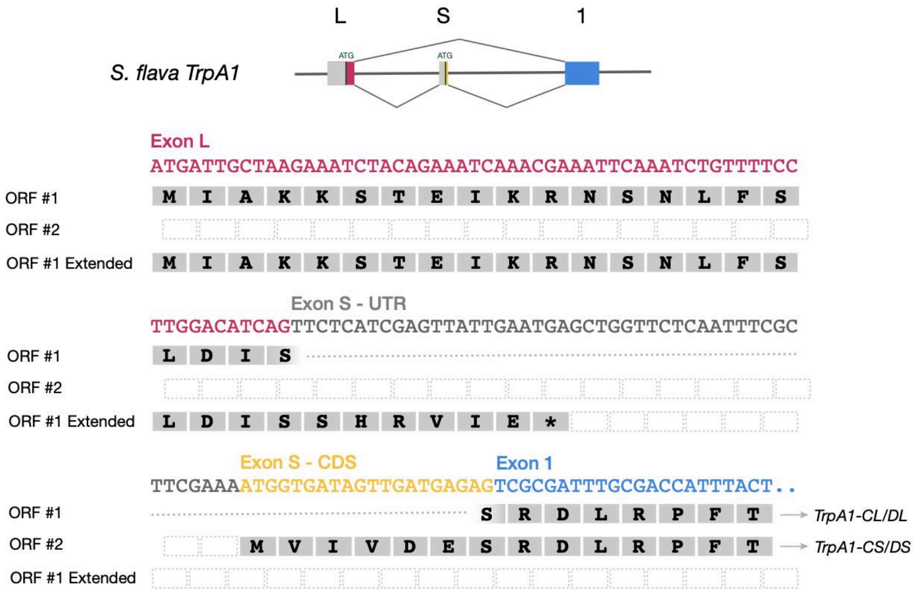

Supplementary Figure S5. Open reading frames (ORFs) of *S. flava TrpA1* splicing isoforms. N-terminal exons and potential ORFs are shown. ORF#1 is likely a splice variant of *TrpA1-CL/DL* splicing isoforms where Exon L directly connects to Exon 1. When Exon S is incorporated into a spliced mRNA, however, ORF#1 would soon encounter a premature stop codon. The longest products would be translated from ORF#2 for *TrpA1-CS/DS* isoforms, which would result in shorter products despite the longer mRNAs. The entire Exon L may be a 5'UTR for the translation of *TrpA1-CS/DS* isoforms.

bioRxiv preprint doi: https://doi.org/10.1101/2025.05.09.653172; this version posted May 15, 2025. The copyright holder for this preprint (which was not certified by peer review) is the author/funder, who has granted bioRxiv a license to display the preprint in perpetuity. It is made available under aCC-BY 4.0 International license.

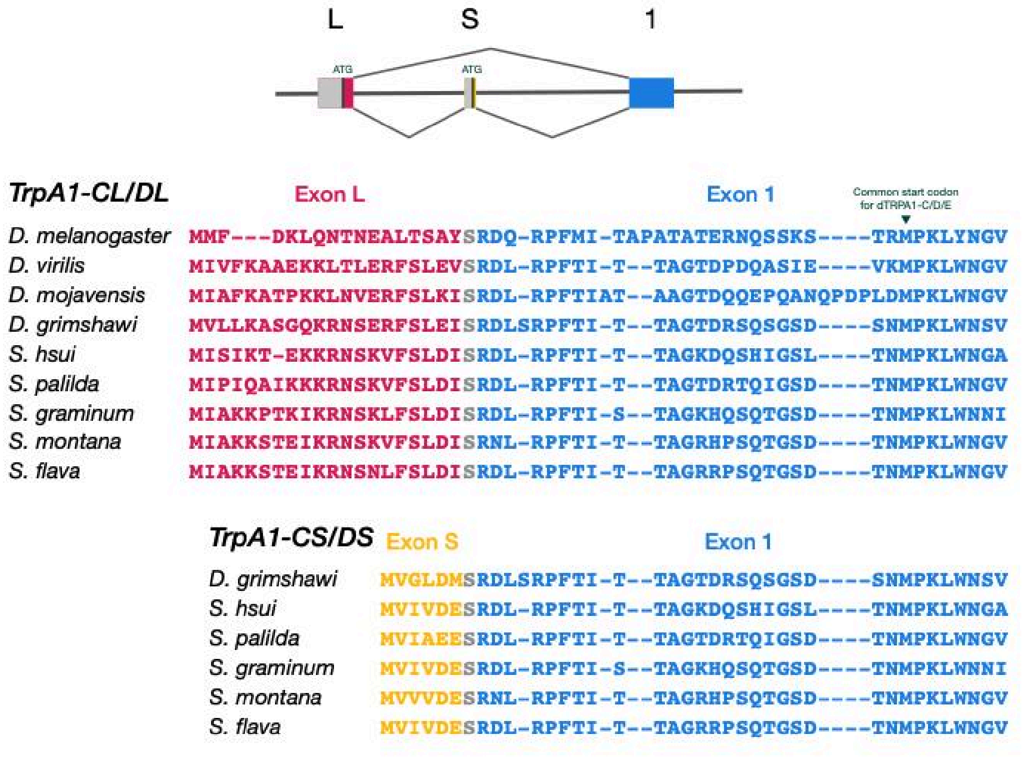

Supplementary Figure S6. N-terminal protein alignments of *TrpA1* splicing isoforms across drosophilids. Note that Exon L and Exon 1 of *D. melanogaster TrpA1* together form a single exon (see Figure S4), the majority of which is usually treated as 5'UTR.

bioRxiv preprint doi: https://doi.org/10.1101/2025.05.09.653172; this version posted May 15, 2025. The copyright holder for this preprint (which was not certified by peer review) is the author/funder, who has granted bioRxiv a license to display the preprint in perpetuity. It is made available under aCC-BY 4.0 International license.

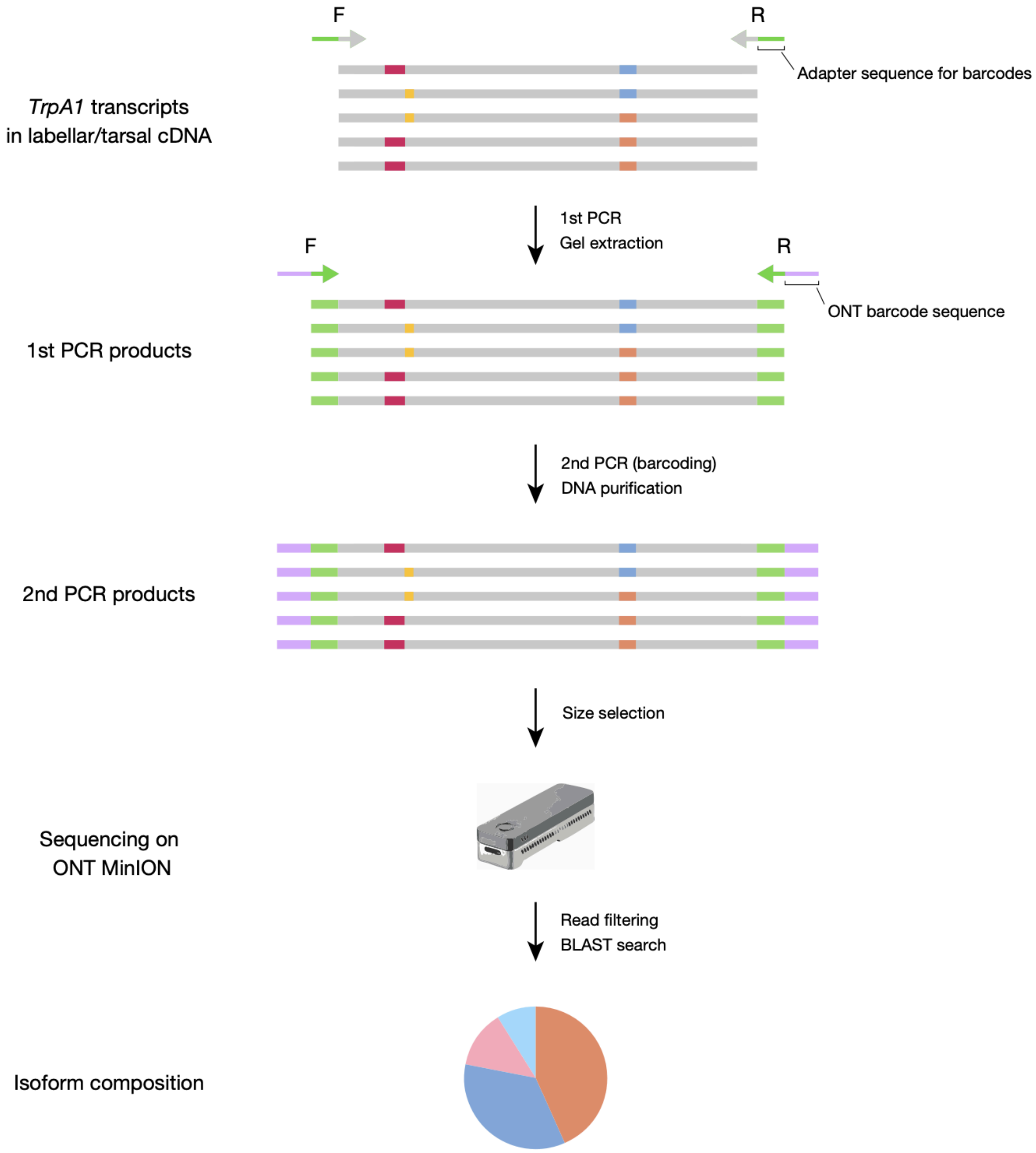

Supplementary Figure S7. A schematic diagram of *TrpA1* amplicon sequencing. Colored regions indicate alternatively spliced exons. An original image for ONT MinION sequencer was obtained from: https://store.nanoporetech.com/us/minion-mk1b-basic-starter-pack.html.

bioRxiv preprint doi: https://doi.org/10.1101/2025.05.09.653172; this version posted May 15, 2025. The copyright holder for this preprint (which was not certified by peer review) is the author/funder, who has granted bioRxiv a license to display the preprint in perpetuity. It is made available under aCC-BY 4.0 International license.

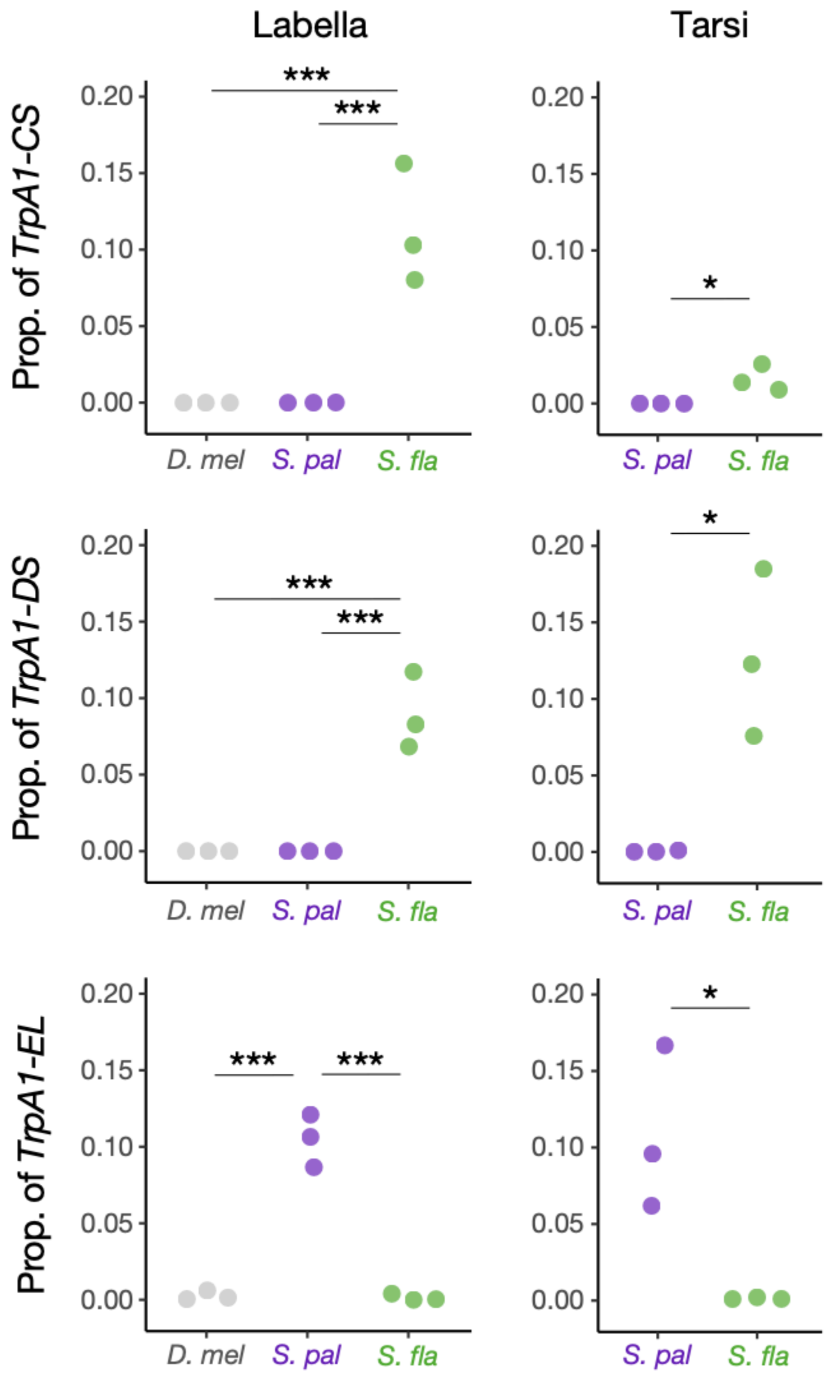

Supplementary Figure S8. Relative abundances of *TrpA1-CL/DL/EL* splicing isoforms. Variation among species (*D. melanogaster* or *D. mel*, *S. pallida* or *S. pal*, and *S. flava* or *S. fla*) was modeled by GLM with Gaussian distribution. Labellar comparisons were further tested by Tukey's post-hoc test with Benjamini-Hochberg correction. *: $p < 0.05$, **: $p < 0.01$, ***: $p < 0.001$.

bioRxiv preprint doi: https://doi.org/10.1101/2025.05.09.653172; this version posted May 15, 2025. The copyright holder for this preprint (which was not certified by peer review) is the author/funder, who has granted bioRxiv a license to display the preprint in perpetuity. It is made available under aCC-BY 4.0 International license.

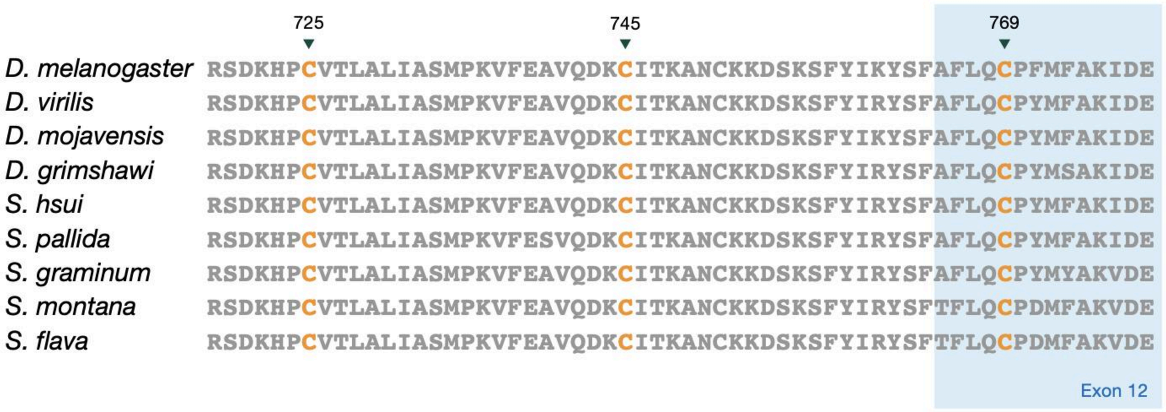

Supplementary Figure S9. Key cysteine residues of TRPA1 are conserved across drosophilids. ITCs and many other TRPA1-activating chemicals are known to form covalent bonds with a few intracellular cysteine residues to trigger gating of the channel. Three important cysteines implicated in human TRPA1 (Hinman et al. 2006) are highlighted. Positions of amino acid residues are based on the order of *S. flava* TRPA1. Note that the cysteine at 769th residue falls on Exon 12, which is an alternatively spliced exon and only present in homologues of TRPA1-A/D.

bioRxiv preprint doi: https://doi.org/10.1101/2025.05.09.653172; this version posted May 15, 2025. The copyright holder for this preprint (which was not certified by peer review) is the author/funder, who has granted bioRxiv a license to display the preprint in perpetuity. It is made available under aCC-BY 4.0 International license.

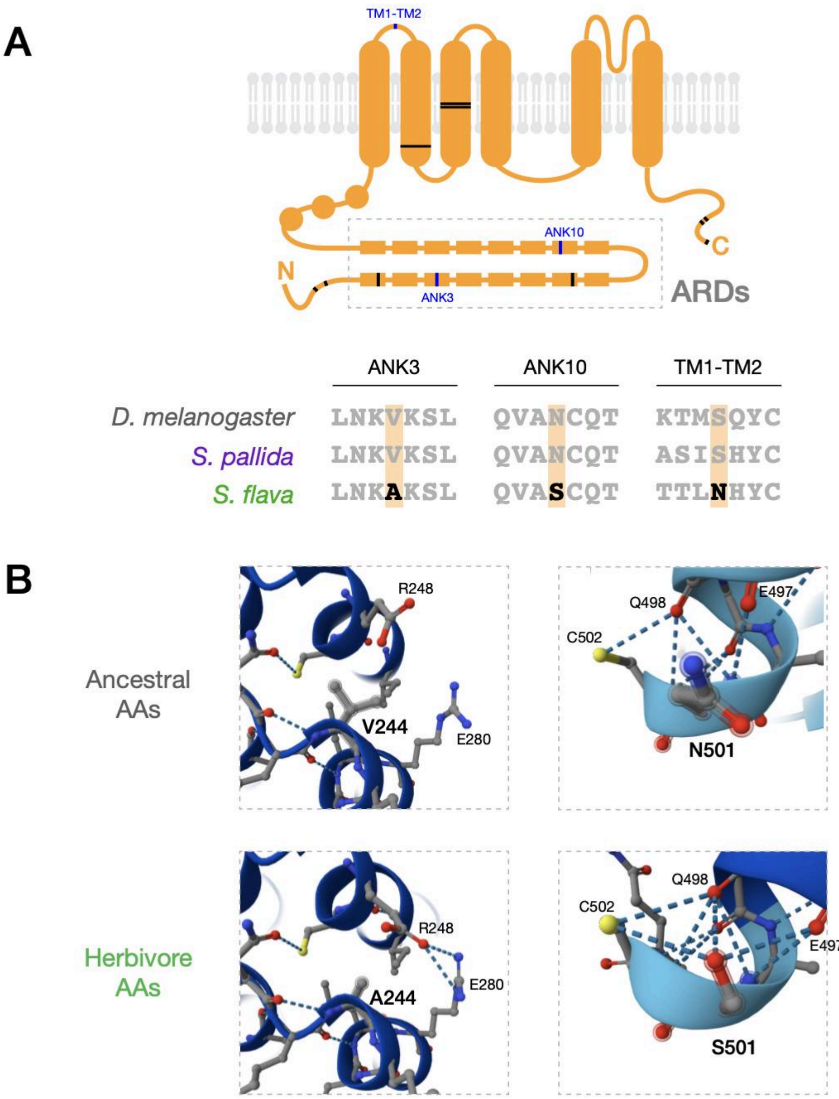

Supplementary Figure S10. Herbivore-specific amino acid substitutions potentially modify the secondary structure of TRPA1. (A) Bars in the TRPA1 protein cartoon highlights the positions of herbivore-specific amino acid substitutions. Blue bars indicate substitutions that could potentially influence local structures of the TRPA1 channel. (B) AlphaFold-led prediction of the secondary structure and hydrogen bonds at 244th (Left, 3rd ANK domain) and 501st (Right, 10th ANK domain) residues of TRPA1 ankyrin repeat domain. Dashed lines indicate predicted hydrogen bonds between adjacent residues. The top row shows predictions with ancestral amino acids (V244, N501), while the bottom row shows those with derived herbivore-specific amino acids (A244, S501).

bioRxiv preprint doi: https://doi.org/10.1101/2025.05.09.653172; this version posted May 15, 2025. The copyright holder for this preprint (which was not certified by peer review) is the author/funder, who has granted bioRxiv a license to display the preprint in perpetuity. It is made available under aCC-BY 4.0 International license.

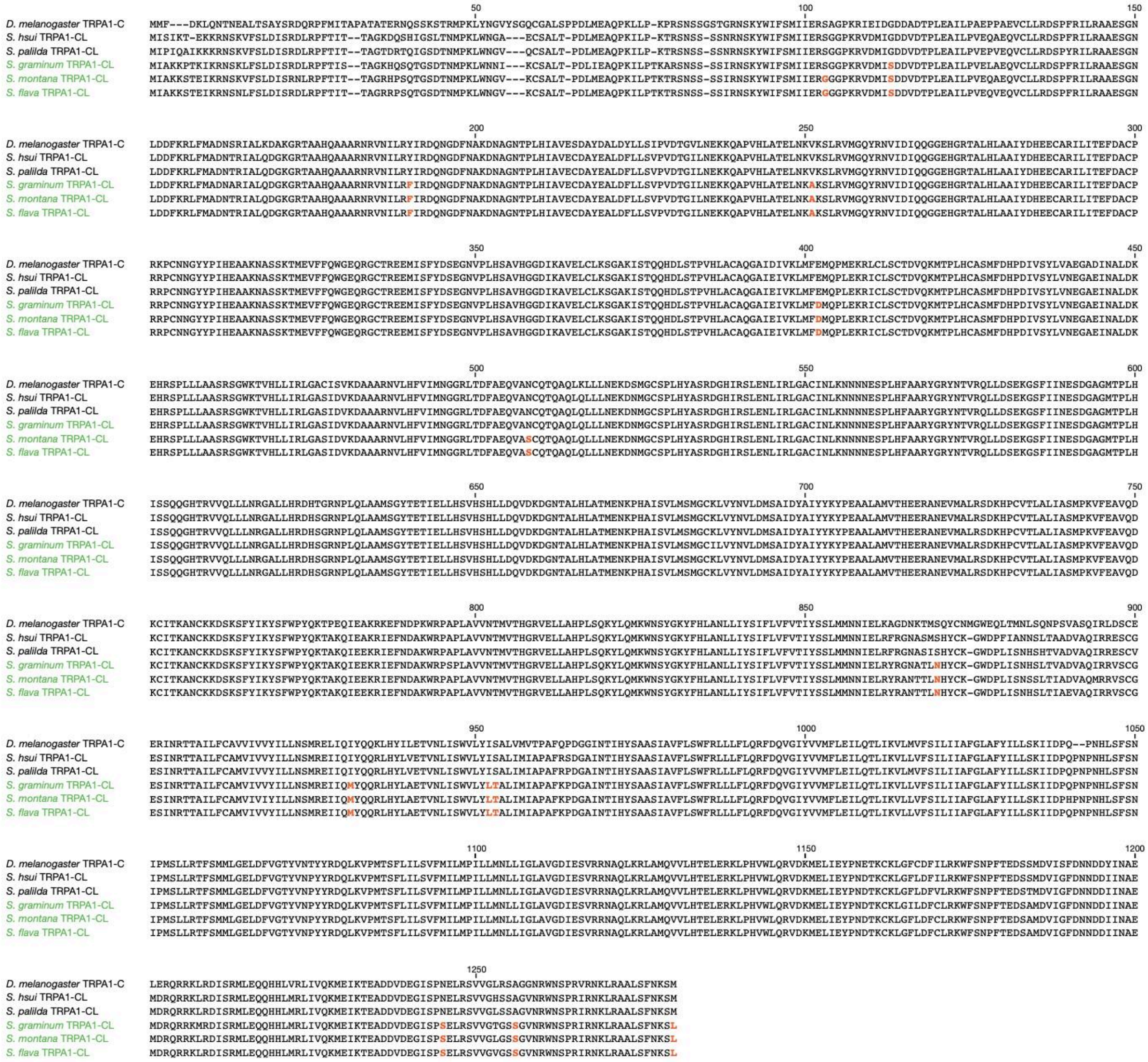

Supplementary Figure S11. A multiple sequence alignment of TRPA1 from six *Drosophila* and *Scaptomyza* species. Green color indicates herbivorous species. Amino acid substitutions specific to herbivores or the mustard-feeding lineage (i.e., *S. montana* and *S. flava*) that are otherwise fixed are indicated in orange.

bioRxiv preprint doi: https://doi.org/10.1101/2025.05.09.653172; this version posted May 15, 2025. The copyright holder for this preprint (which was not certified by peer review) is the author/funder, who has granted bioRxiv a license to display the preprint in perpetuity. It is made available under aCC-BY 4.0 International license.

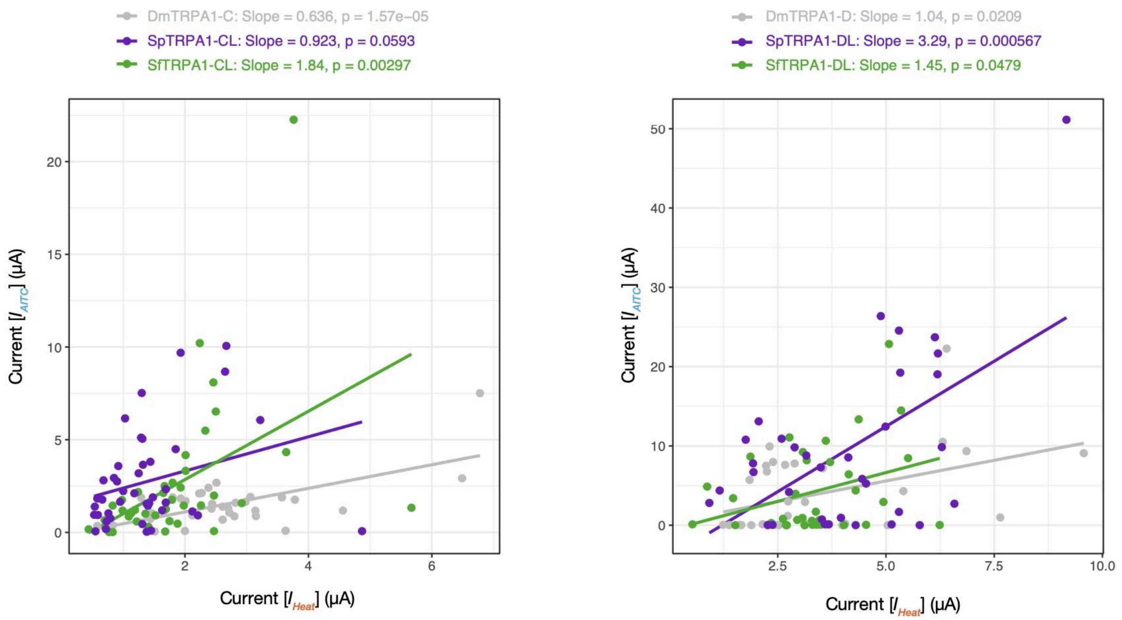

Supplementary Figure S12. Correlations between raw heat-evoked and AITC-evoked currents. Each dot represents a single oocyte. (Left) TRPA1-CL and (Right) TRPA1-DL splicing isoforms. Data from all AITC concentrations were pooled for each splicing isoform.

bioRxiv preprint doi: https://doi.org/10.1101/2025.05.09.653172; this version posted May 15, 2025. The copyright holder for this preprint (which was not certified by peer review) is the author/funder, who has granted bioRxiv a license to display the preprint in perpetuity. It is made available under aCC-BY 4.0 International license.

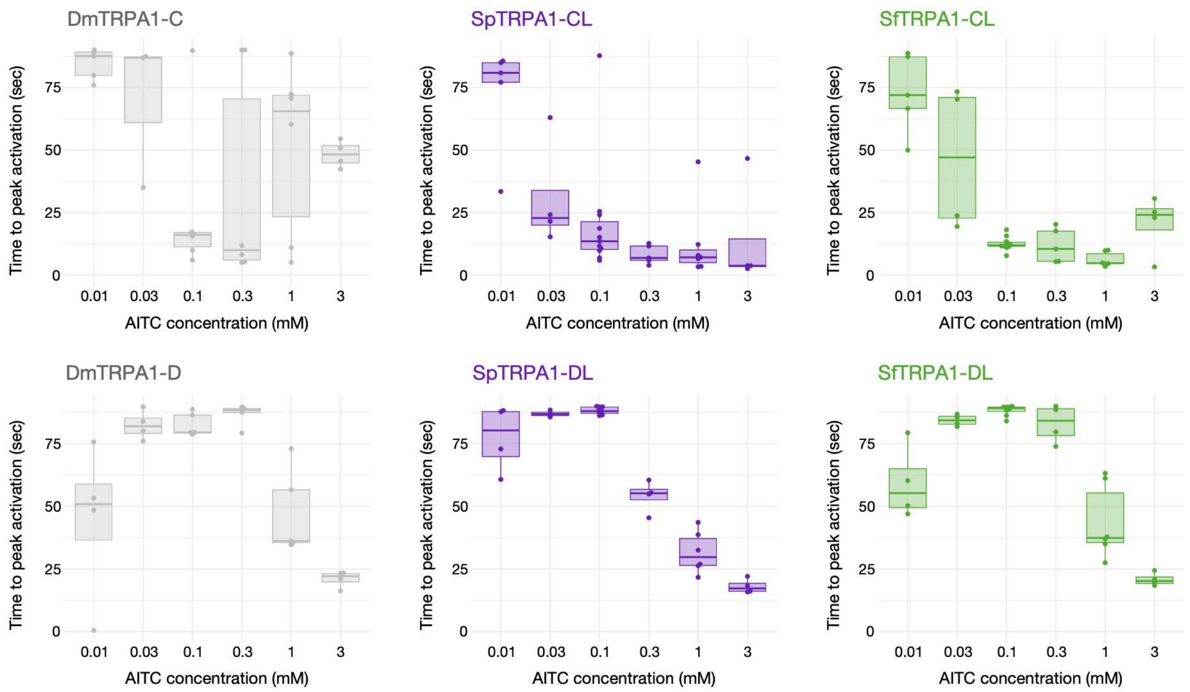

Supplementary Figure S13. Times to reach peak activation in six canonical TRPA1 splicing isoforms. Recordings were carried out within a 90-second AITC stimulation window, across the concentration series from 0.001 mM to 3 mM AITC. Note that the smallest concentration (0.001 mM AITC) was omitted from this analysis due to the lack of observable chemical-invoked currents. Overall, TRPA1-CL(C) isoforms tended to peak faster than TRPA1-DL(D) splicing isoforms.

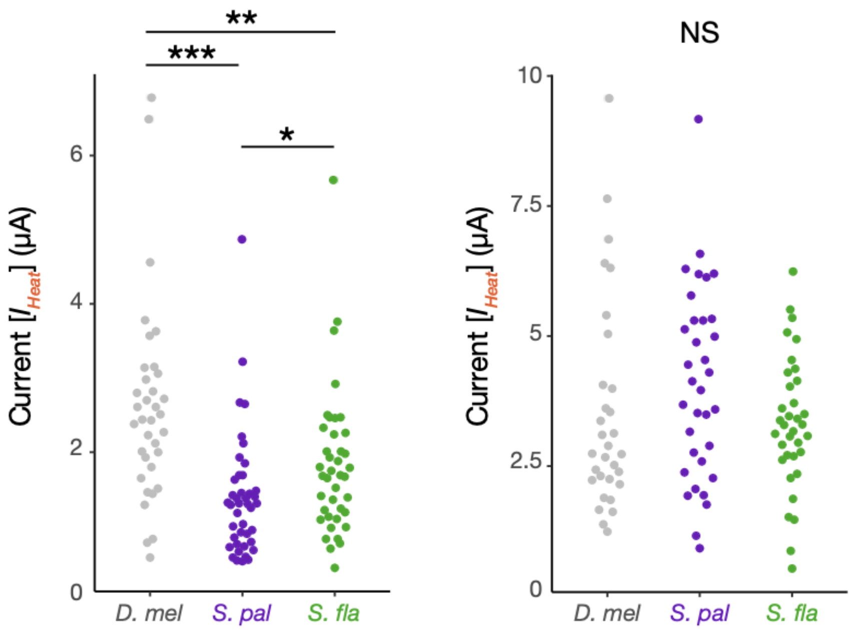

Supplementary Figure S14. Comparisons of raw heat-evoked currents (I_{Heat}). (Left) TRPA1-CL and (Right) TRPA1-DL splicing isoforms, tested by Kruskal-Wallis test and post-hoc Conover's test with Benjamini-Hochberg correction. *: $p < 0.05$, **: $p < 0.01$, ***: $p < 0.001$.

bioRxiv preprint doi: https://doi.org/10.1101/2025.05.09.653172; this version posted May 15, 2025. The copyright holder for this preprint (which was not certified by peer review) is the author/funder, who has granted bioRxiv a license to display the preprint in perpetuity. It is made available under aCC-BY 4.0 International license.

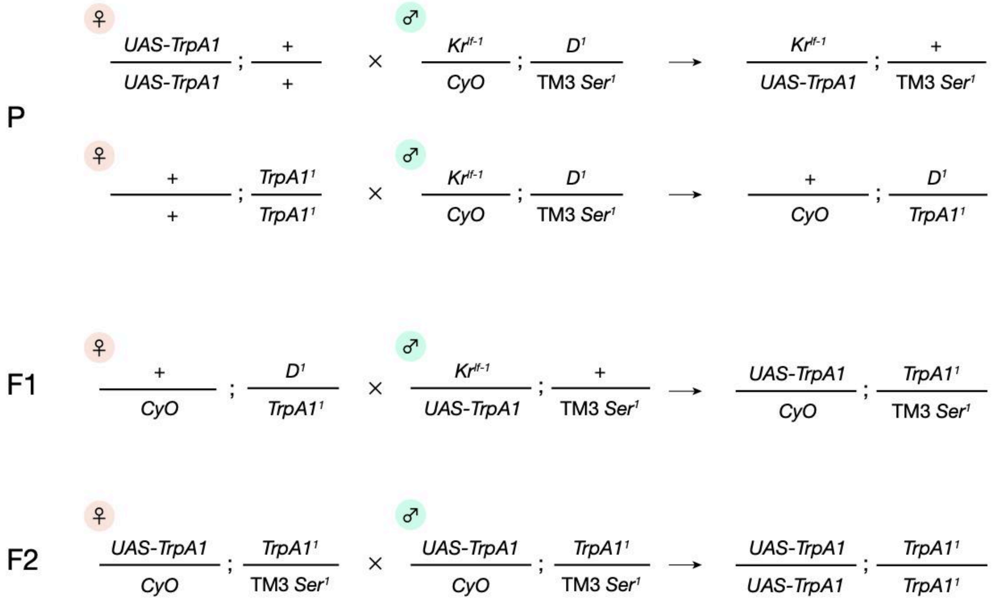

Supplementary Figure S15. Crossing schemes to obtain double homozygotes of *UAS-TrpA1* and *TrpA1^{1}* alleles. A double balancer line (*Kr^{If-1}/CyO*;*D^{1}*/TM3 *Ser^{1}*) was utilized to merge two alleles. Only second and third chromosomes are shown. *Gr66a-GAL4*;*TrpA1^{1}* homozygotes were also obtained in the same procedure.

bioRxiv preprint doi: https://doi.org/10.1101/2025.05.09.653172; this version posted May 15, 2025. The copyright holder for this preprint (which was not certified by peer review) is the author/funder, who has granted bioRxiv a license to display the preprint in perpetuity. It is made available under aCC-BY 4.0 International license.

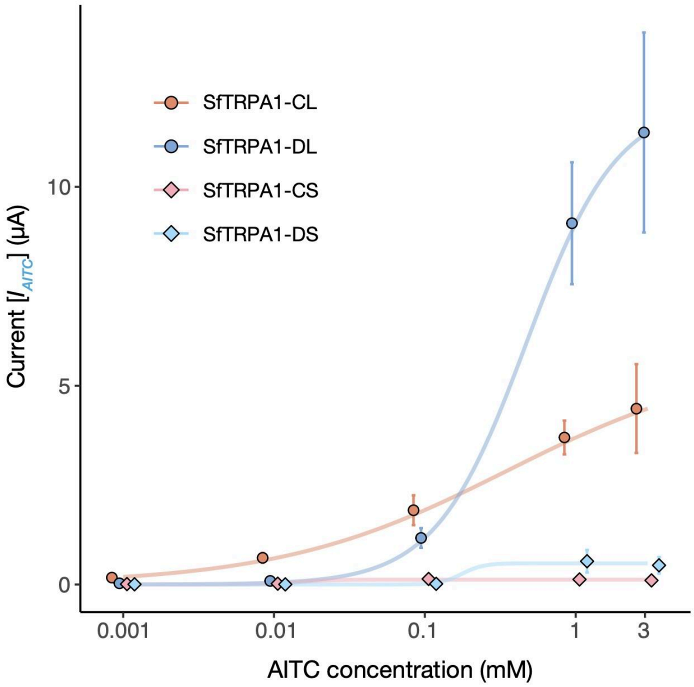

Supplementary Figure S16. Dose-response curves of the four SfTRPA1 isoforms. Note that the prediction of dose-response was performed on raw current values (I_{AITC}), instead of normalized values (I_{AITC}/I_{Heat}), because weak heat activation of the inactive isoforms prevented proper normalization. n = 3-12 per group.
